# Low Copper and High Manganese Levels in Prion Protein Plaques

**DOI:** 10.3390/v5020654

**Published:** 2013-02-11

**Authors:** Christopher J. Johnson, P.U.P.A. Gilbert, Mike Abrecht, Katherine L. Baldwin, Robin E. Russell, Joel A. Pedersen, Judd M. Aiken, Debbie McKenzie

**Affiliations:** 1 USGS National Wildlife Health Center, 6006 Schroeder Road, Madison, WI 53711, USA; E-Mails: cjjohnson@usgs.gov (C.J.J.); rerussell@usgs.gov (R.E.R); 2 Department of Physics, University of Wisconsin-Madison, 1150 University Avenue, Madison, WI 53706, USA; E-Mails: pupa@physics.wisc.edu (P.U.P.A.G.); mike.abrecht@comet.ch (M.A.); 3 Program in Cellular & Molecular Biology, University of Wisconsin-Madison, 425-G Henry Mall Madison, WI 53706, USA; E-Mail: klbaldwin@wisc.edu (K.L.B.); 4 Department of Soil Science, University of Wisconsin-Madison, 1525 Observatory Dr., Madison, WI 53706, USA; E-Mail: joelpedersen@wisc.edu (J.A.P.); 5 Centre for Prions and Protein Folding Diseases, AFNS, University of Alberta, Edmonton, Alberta, T6G 2M8, Canada; E-Mail: judd.aiken@ualberta.ca (J.M.A.); 6 Centre for Prions and Protein Folding Diseases, Department of Biological Sciences, University of Alberta, Edmonton, Alberta, T6G 2M8, Canada; E-Mail: debbie.mckenzie@ualberta.ca (D.M.)

**Keywords:** transmissible spongiform encephalopathy, X-ray photoemission, copper, manganese, prion, brain

## Abstract

Accumulation of aggregates rich in an abnormally folded form of the prion protein characterize the neurodegeneration caused by transmissible spongiform encephalopathies (TSEs). The molecular triggers of plaque formation and neurodegeneration remain unknown, but analyses of TSE-infected brain homogenates and preparations enriched for abnormal prion protein suggest that reduced levels of copper and increased levels of manganese are associated with disease. The objectives of this study were to: (1) assess copper and manganese levels in healthy and TSE-infected Syrian hamster brain homogenates; (2) determine if the distribution of these metals can be mapped in TSE-infected brain tissue using X-ray photoelectron emission microscopy (X-PEEM) with synchrotron radiation; and (3) use X-PEEM to assess the relative amounts of copper and manganese in prion plaques *in situ*. In agreement with studies of other TSEs and species, we found reduced brain levels of copper and increased levels of manganese associated with disease in our hamster model. We also found that the *in situ* levels of these metals in brainstem were sufficient to image by X-PEEM. Using immunolabeled prion plaques in directly adjacent tissue sections to identify regions to image by X-PEEM, we found a statistically significant relationship of copper-manganese dysregulation in prion plaques: copper was depleted whereas manganese was enriched. These data provide evidence for prion plaques altering local transition metal distribution in the TSE-infected central nervous system.

## 1. Introduction

Transmissible spongiform encephalopathies (TSEs, prion diseases) are fatal neurodegenerative diseases that affect humans and other mammals. The primary component of the infectious TSE agent is a misfolded conformer of the prion protein denoted PrP^Sc^, which typically accumulates in diseased nervous tissue, often as large plaques [1]. The abnormally folded PrP^Sc^ molecule is derived from a normal, cellular isoform of the prion protein (PrP^C^). Both PrP^C^ and PrP^Sc^ have identical amino acid sequences and covalent posttranslational modifications and differ only in secondary and higher structure. The prion protein amino acid sequence possesses an octapeptide repeat region with metal binding capacity at the *N*-terminus of the protein. Evidence suggests that PrP^C^ complexes copper with a dissociation constant, *K_d_*, in the nanomolar range and other metals, most notably manganese, may also be bound [2,3]. Complexation of Mn^2+^ by PrP^C^ may occur within the octapeptide repeat region or a second site at histidine 95 and occurs with a *K_d_* in the micromolar range [3]. Loading PrP^C^ with manganese induces conformational changes in the protein reminiscent of some of the characteristics of PrP^Sc^, such as increased β-sheet content, aggregation and increased protease resistance [4]. Analysis of the metal occupancy in PrP^Sc^ in brain homogenates from TSE-infected mice indicates reduced levels of copper and increased quantities of manganese compared to PrP^C^ from control brains, suggesting that metal binding may contribute to the mechanism of PrP^C^-PrP^Sc^ conversion [5,6].

Dysregulation of copper-manganese homeostasis has been hypothesized to contribute to, or even cause, TSE-related pathology and death [7,8], and manipulation of metal levels has been reported to modestly prolong the incubation period in scrapie-infected mice [9,10,11]. Brains of TSE-infected humans and animals have reduced levels of copper and increased levels of manganese [5,6,12,13,14]. Presumably, metal binding by PrP^Sc^ contributes to these alterations in brain metal concentrations, but information on the spatial distribution of metal in, or near, prion plaques *in situ* has not been reported. Synchrotron radiation X-ray photoemission electron microscopy (X-PEEM) is a sensitive spectromicroscopy method capable of mapping most elements on a micron scale at sensitivity down to the parts per billion range [15,16,17]. In this study, we assess bulk copper and manganese concentrations in healthy and TSE-infected hamster brain homogenates and use X-PEEM to localize the distribution of copper and manganese in prion plaques.

## 2. Materials and Methods

Animals used in this study were cared for according to institutionally approved protocols. For all experiments, hamsters were infected intracerebrally with the HY strain of hamster-adapted transmissible mink encephalopathy agent [18]; the controls were sham-inoculated with saline. Brains were collected at clinical disease (~62 days post-infection) and stored at -80 °C until use. For metal content experiments, brains from hamsters clinically affected with HY strain or age-matched, sham-inoculated controls were homogenized in sterile, distilled, deionized H_2_O using Dounce homogenizers. Nitric acid (Trace Metal Grade, Fisher Scientific) was added to a final concentration of 50% and samples were heated at 100 °C for 2 hours or until the solution clarified. All glass and plastic-ware for this procedure was soaked in 10% nitric acid for 24 hours prior to use, and all samples were run in parallel. Analysis of copper and manganese concentrations was performed by the University of Wisconsin Soil and Plant Analysis Laboratory using inductively coupled plasma-mass spectrometry (ICP-MS) following their standard protocols. Copper and manganese concentrations are reported on a wet weight basis.

For X-PEEM experiments, brains from two clinically-affected HY-infected hamsters and two age-matched, sham-inoculated controls were removed and embedded in OCT Tissue Tek compound (Miles Scientific). Two consecutive coronal cryosections of brainstem (7 μm) were cut for each animal analyzed. The first section was investigated by immunohistochemistry, and the second section was prepared for X-PEEM. The first section was placed on a glass microscope slide, immunostained following a published protocol with monoclonal anti-prion protein antibody 3F4 to label PrP^Sc^ [19], and a composite image of the stained tissue from the first section was generated to identify regions of interest for X-PEEM analysis. The second section was placed on an inert silicon wafer for X-PEEM analysis and ashed with UV-ozone at ambient temperature for two weeks to remove carbon, thereby increasing the relative abundances of metals [20] and reducing prion infectivity [21].

Brain tissue samples were analyzed by X-PEEM using the spectromicroscope for photoelectron imaging of nanostructures with X-rays (SPHINX; Elmitec), installed on the HERMON beamline at the Synchrotron Radiation Center at the University of Wisconsin [15]. Briefly, samples were mounted vertically and illuminated with monochromatic soft X-rays either on or off of the L-edge for each element. The photoelectrons emitted by the sample were accelerated toward an electron optics column and onto a phosphor screen. The chamber was held at ultrahigh vacuum (10^−10^ Torr). The surface image of the sample was acquired by a slow-scanning air-cooled CCD camera. Maps of elements were prepared as we have previously described [16]. Pseudocoloring and image mergers were performed in Adobe Photoshop.

Images were imported as tiff files into Program R [22] using the rtiff package [23]. This function creates raster files of the image where the colors are quantified and scaled from 0-1. We compared copper and manganese distributions and estimated Pearson’s correlation coefficient for each metal for each region. Additionally, we conducted a regression model using the glm function in R to estimate the relationship between intensity of color on the copper and manganese images, while accounting for potential differences between regions. 

## 3. Results and Discussion

Changes in brain metal concentrations during the course of a TSE infection are thought to be due to the replacement of copper for manganese in PrP^Sc^, and metal imbalances of copper and manganese have been noted for TSEs of mice, cattle, sheep and humans [5,6,12,13,14]. Additionally, Kim *et al.* observed increased manganese in brain homogenates of hamsters clinically-affected with the 263K strain of hamster TSE, but did not report findings for copper [24]. Using ICP-MS, we found that HY-infected brains had reduced copper and increased manganese content compared to age-matched controls, consistent with other TSEs in other host species. Copper concentrations in HY-infected brains were 5.3 ± 0.4 μg∙g_tissue_^-1^, significantly lower than in brains of healthy controls, which measured 6.8 ± 0.2 μg∙g_tissue_^-1^ (*t* = 3.95, df = 4, *p* = 0.017). Manganese concentrations in HY-infected and uninfected brains were 0.57 ± 0.04 and 0.47 ± 0.01 μg∙g_tissue_^-1^ of tissue, respectively, and differed significantly (*t* = 6.25, df = 4, *p* = 0.003). Infection of hamsters with HY TSE agent changes brain copper and manganese levels to values nearly identical to those found in mice infected with the RML-strain of TSE agent [6]. Our results indicate that the HY TSE agent in the hamster model is an appropriate choice for studying TSE-associated metal dysregulation and support the hypothesis that metal dysregulation is a phenomenon that spans multiple TSEs. 

*In situ* investigation of copper and manganese levels in uninfected control brainstem tissues by X-PEEM revealed that manganese was below the limit of detection and that copper was present at detectable levels and homogenously distributed when imaged with ~1 μm spatial resolution (data not presented). Since copper was detectable, we proceeded with X-PEEM imaging of HY-infected brainstem tissue. Using X-PEEM, we analyzed plaques in the brainstem of HY TSE-infected hamsters. Prion plaques vary in size, but plaques in the brainstem of hamsters infected with this strain of TSE frequently span >10 μm in three dimensions [19], facilitating analysis of the same plaque across subsequent sections.

**Figure 1:**
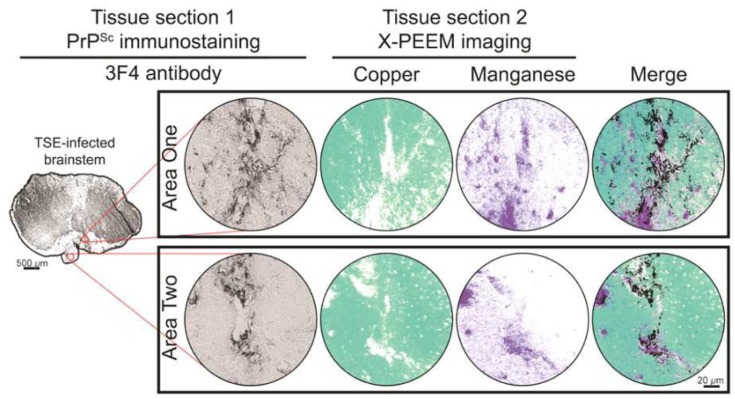
A coronal section (Tissue section 1) of a brainstem from a HY TSE-infected hamster was immunostained for PrP^Sc^ with monoclonal antibody 3F4. Areas with PrP^Sc^ deposits (as indicated by a black reaction product) larger than 7 µm were identified for analysis by X-PEEM on Tissue section 2, a directly adjacent tissue section. In Tissue section 2, copper and manganese distributions were assessed by X-PEEM in each area of PrP^Sc^ deposition. The presence or absence of copper is indicated by aqua pseudocoloring or white, respectively. Purple pseudocoloring or white represents the presence or absence of manganese, respectively. Superimposing PrP^Sc^ immunostaining micrographs with copper and manganese images (Merge) indicates the spatial distribution of all signals. Scale bars are labeled with the appropriate lengths for the corresponding images.

We identified regions of potential interest for X-PEEM analysis in slides immunostained with an anti-prion protein antibody (Figure 1). We chose regions containing prion plaques that were several times larger than 7 μm so that they would likely extend into the next section. The PrP^Sc^ staining was generally perineuronal. After identifying regions of interest on the PrP^Sc^-stained slide, we imaged the corresponding regions in the subsequent tissue section with X-PEEM (Figure 1, Areas 1 and 2). In the X-PEEM images, we found the background levels of copper were fairly homogenous, but also identified large (>20 μm) and small (~1 μm) structures where copper was below the limit of detection. Similar results were observed in two different regions of the brainstem for two separate animals. All samples are from the brainstem; area 1 corresponds to reticular nuclei of the brainstem and area 2 is the inferior olivary nucleus. Results from one animal are shown.

By comparing the intensities of 20 areas inside and outside of the copper-depleted structures, we found that these Cu-depleted structures had lower copper concentrations than surrounding tissue by a factor of 25.05 (95% confidence interval: 25.45 to 26.65). Unlike uninfected control tissue, manganese was detectable in HY-infected tissue and in large and small structures where it was inversely correlated with copper distribution. Using the same comparative method of calculation used for copper, manganese levels in these structures was increased by a factor of 24.30 (95% confidence interval: 21.33 to 27.27) relative to tissue not involved with these structures. When compared to micrographs of the previous tissue section stained with an antibody recognizing the prion protein (Figure 1), the morphology of structures lacking copper and enriched for manganese visually matched the prion plaques from the previous section.

Pearson’s product-moment correlation of -0.25 (p-value <0.001) indicated a negative relationship between the copper and manganese images (i.e., where one image was lighter the other image was darker). Regression coefficients revealed a statistically significant relationship between values of manganese and copper (estimated coefficient for copper [-0.44, p-value < 0.001]). The estimated coefficient indicates that for every 0.10 unit increase in the image intensity for copper there is a corresponding 0.044 decline in the intensity of the manganese image (where light color=0 and dark color=1). Models also indicated an effect of individual region, [estimated coefficient=0.05, p-values < 0.001], indicating that the decline in color intensity on the manganese image was approximately 0.005 less for one region versus the other for every 0.10 unit increase in copper.

While X-PEEM does not permit quantitative analysis of metal concentrations, our data indicate significantly increased manganese and diminished copper content in prion plaques compared to surrounding tissue. These data further confirm that PrP^Sc^ exists in a low copper, high manganese state *in vivo* and supports the hypothesis (4) that, while copper is important for the function of PrP^C^; the conversion of PrP^C^ to PrP^Sc^ may require manganese. 

## 4. Conclusions

Our study provides the first *in situ* analysis of copper and manganese levels in prion plaques and illustrates the specific localization and sizes of copper-poor and manganese-rich regions. Whether these changes to brain metal content are a cause or consequence of TSE infection, and the extent that these altered metal concentrations contribute to the tissue damage and cell loss remains to be elucidated. This study has investigated plaques in only one brain region and only two of many potential metals that may influence TSE pathogenesis. A comprehensive study designed to assess the distributions of iron [25], zinc [26,27] and other metals, as has recently been published for brains of prion protein knock-out and overexpressing mice [28], is warranted. The near equivalence of the reduction in copper and increase in manganese in plaques is consistent with a 1:1 replacement of the metals in the PrP^Sc^ molecule and suggest a role for these metals in TSE neuropathology. Our data are also consistent with previous studies, both *in vivo* and *in vitro,* that suggest a role for manganese in prion infections and our data demonstrates that increased levels of manganese, with concommitent decreased levels of copper, can be localized to brain regions containing prion plaques.
